# A decline in glycolytic ATP production is the fundamental mechanism limiting lifespan; species with an optimal rate of decline over time survived

**DOI:** 10.18632/aging.206356

**Published:** 2026-02-23

**Authors:** Akihiko Taguchi, Yuka Okinaka, Carsten Claussen, Sheraz Gul

**Affiliations:** 1 Department of Regenerative Medicine Research, Foundation for Biomedical Research and Innovation at Kobe, Hyogo 650-0047, Japan; 2 Fraunhofer Institute for Translational Medicine and Pharmacology ITMP, Discovery Research ScreeningPort, Hamburg 22525, Germany

**Keywords:** hypothesis, aging, glycolytic ATP production, lifespan, *Heterocephalus glaber*

## Abstract

Glycolytic ATP production declines with age, contributing to common aging phenotypes such as reduced cell division and impaired DNA & mitochondria repair. Notably, immortal cells exhibit a metabolic profile characterized by sustained, highly active glycolytic ATP production. A key unresolved question is the underlying mechanism driving the gradual decline in glycolytic ATP production during natural aging. We have found that this can be explained by the concept that a decline in glycolytic ATP production was crucial for survival of species, and only those species with an optimal rate of reduction in glycolytic ATP production over time were selected and persisted through generational changes. Sexual reproduction generates new combination of gene pairs with abundant DNA mutations during meiosis, which provides significant advantages in adapting to environmental changes and competence over other species. However, the population of species is limited because of finite food supply in the natural world. The shift from glycolysis to aerobic metabolism increases energy efficiency and the increased energy efficiency in parent generation benefits the species by enhancing survival of parent generation at starvation conditions and limited food allocation to the offspring generation. This conceptual framework can explain the finite lifespans of organisms, significant variations in lifespan across species, cellular immortality of cancer cells, and the exceptionally long life of the naked mole rat (*Heterocephalus glaber*). Although questions remain, this concept offers new insights into the biology of aging and potential strategies for rejuvenation therapies for humans.

## INTRODUCTION

A number of hypotheses have been proposed to explain the causes of aging, including DNA errors, telomere shortening, epigenetic modifications, and mitochondrial damages. However, these theories remain largely inconclusive and inconsistent, and more importantly, they lack clinical validation and may represent effects rather than causes of aging. Recent translational and clinical studies have indicated that hematopoietic stem cells (HSC) promote vascular regeneration by stimulating glycolytic ATP production via gap junctions [1]. Regeneration and aging can be viewed as interconnected processes, prompting us to investigate the role of glycolytic ATP production in aging.

### The need for a new concept for aging

First, why do animals not have an eternal lifespan? Animals possess sophisticated systems that, in many species, appear capable of supporting immortality. Second, why do lifespans vary considerably among species despite similarities in genetic makeup, specifically the central dogma linking DNA, RNA, and protein synthesis, which warrants a molecular explanation? For example, elephants live thirty times as long as mice. Third, why can cancer cells extensively proliferate with abnormal longevity, resulting in unchecked growth driven by gene mutations? For example, the anti-apoptotic gene *p53* often loses function in tumors, enhancing glycolysis [2] and enabling cells to evade death [3]. However, loss-of-function mutations alone do not explain how cells avoid accumulating mitochondrial damage and DNA errors over time. Fourth, why does an exceptional animal, such as the naked mole rat (*Heterocephalus glaber*), lack an innate lifespan? These fundamental questions indicate the necessity for a new concept to explain aging.

Given that cellular regeneration and aging may represent two sides of the same coin [4], a unified concept reconciling their underlying causes is necessary. This is exemplified by HSC transplantation in patients with limb ischemia [5], where HSC-mediated angiogenesis serves as the primary mechanism. Specifically, interactions between gap junctions and direct cellular contacts activate hypoxia-inducible factor-1α (HIF-1α) in damaged endothelium, increasing glycolytic ATP production [1].

### The role of glycolysis

Significant differences between ATP production by glycolysis and oxidative phosphorylation include the quantity produced, production speed, and functional roles. Glycolytic ATP production is approximately 100 times faster than oxidative phosphorylation [6]. ATP from glycolysis supplies rapid energy during acute demands, while oxidative phosphorylation supports basal/homeostatic cellular energy needs [7]. Glycolysis plays important role in cell division [8] and DNA repair [9]. Additionally, the glycolysis activator HIF-1α promotes mitochondria repair through mitophagy [10]. These findings suggest that decreased glycolytic ATP production during aging may underline various age-related symptoms. Immortal cells exhibit a metabolic profile characterized by highly active glycolytic ATP production and HIF-1α activation, even in oxygen-rich conditions [6]. The oncogene, *c-Myc*, is upregulated in various tumors, increasing glycolytic flux [11]. Immortal cells typically exhibit active mitosis and mitophagy, with aerobic glycolysis playing a crucial role in immortality [12].

### A simple unifying concept underlying aging

**Figure 1:**
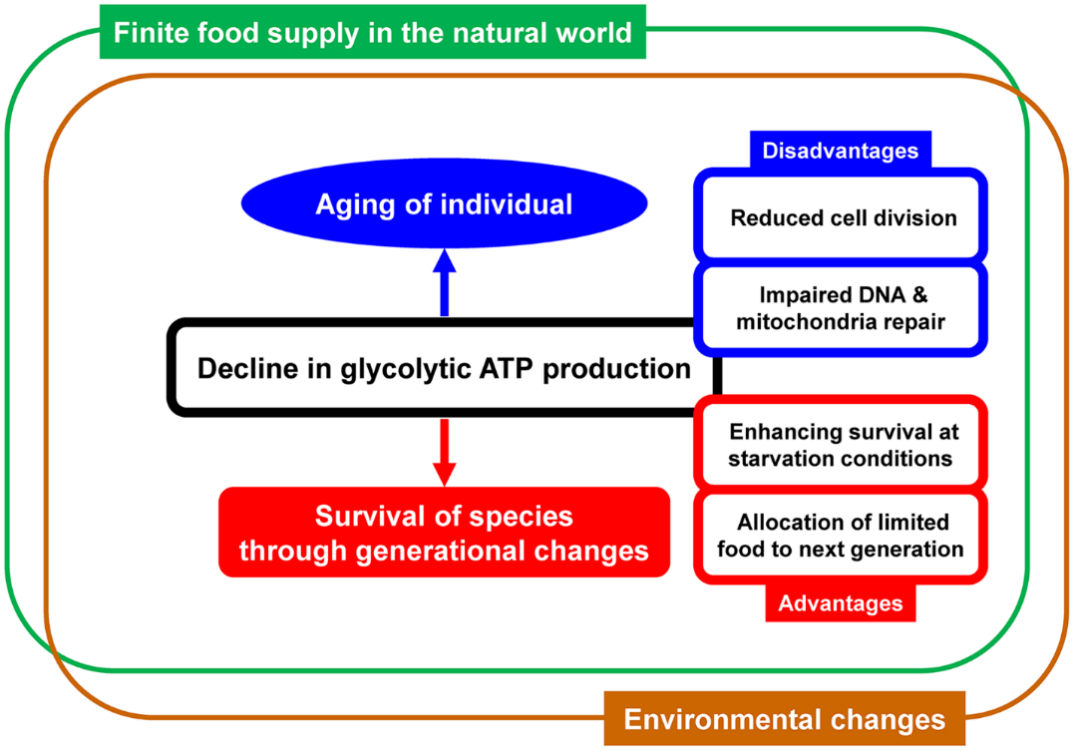
**Schematic representation of the simple concept linking aging and glycolytic ATP production.** Decline in glycolytic ATP production over time has both disadvantages and advantages. Various questions about aging can be explained by the concept that species that happened to have an optimal rate of decline were selected and survived through generational changes.

Populations of species cannot grow infinitely, and one of the major limiting factors in natural world is food supply [13]. The shift from glycolysis to aerobic metabolism increases energy efficiency [14], benefiting individual survival during food shortages [15], which can be caused by environmental changes or emergence of competitors for the food. Additionally, altruism in food sharing for offspring is known in a wide variety of species, including non-human primates [16], bats [17], and insects [18]. These findings indicate that reduced glycolytic ATP production with aging can benefit the species by enhancing survival of parent generation at starvation conditions and allocating food to offspring generation in natural world where food supply is limited (Figure 1).

However, glycolytic ATP production is homeostatically maintained through various feedback mechanisms [19], yet the processes controlling its gradual decline over years remain unclear, despite being observed in nearly all species. Programmed mechanisms that gradually reduce glycolytic ATP production over years with precisely regulated speed would require significant amount of ATP that can contradict the merit of energy saving, necessitating a paradigm shift to explain this phenomenon. The simple explanation is that only species that happened to have an optimal rate of reduction in glycolytic ATP production over time were selected and survived through generational changes. Generational changes with sexual reproduction provide not only new combination of gene pairs but also generate abundant mutations in the process of meiosis, such as meiotic recombination [20]. Natural selection is a mechanism of evolution and organisms that are more adapted to their environment survive [21] and species with wide variety of genes prepared by sexual reproduction have significant advantages for survival by adapting to environmental changes and outcompeting other species [22]. The optimal rate of glycolytic ATP decline for survival varies among species and depends on factors such as environment, competition, maturation time, and body size. This concept clarifies the significant differences in aging rates and lifespans across species despite largely conserved biological components. This is exemplified by the naked mole rat (*H. glaber*), an exceptionally long-lived species that lives underground where there are few environmental changes and predators, and maintains unrestrained glycolytic flux and ATP supply to adapt to underground life with low oxygen levels [23].

### Links between glycolytic ATP decline and aging phenotypes

Aerobic glycolysis in neurons is known to protect against oxidative damage [24]. Brain glucose metabolism deteriorates in a progressive in age-related neurodegenerative diseases, such as Alzheimer’s disease [25–27]. Notably, impaired brain glucose metabolism in Alzheimer’s disease is not solely a consequence of neuronal dysfunction, but the progressive decline in glycolytic ATP production precedes decline in ATP production by oxidative phosphorylation [28]. In the other neurodegenerative diseases like Parkinson’s disease, enhancing glycolysis by terazosin, which enhances the activity of phosphoglycerate kinase 1 and stimulating glycolysis, had been shown to attenuate progression in models and clinical databases [29]. Peritubular endothelial cell dropout leading to microvascular rarefaction is a common pathology of chronic kidney disease (CKD) [30]. Peritubular endothelial cells have a hypoglycolytic metabolism in CKD and restoration of glycolysis in peritubular endothelial cells by overexpressing 6-phosphofructo-2-kinase/fructose-2, 6-bisphosphatase had been shown to attenuate microvascular rarefaction and kidney fibrosis [30]. Furthermore, the activation of AKT1, which has a potential to activate glycolysis [31], has been shown to suppress sarcopenia, that is a progressive decline in glycolytic fast-twitch muscle with aging [32]. These reports indicate the close link between glycolytic ATP decline and specific aging phenotypes in various organs and the actual speed of the decline may reflect the precision of homeostatic maintenance mechanisms. However, a hypothesis that can explain a phenomenon or relationship does not necessarily imply its validity. The validity of the hypothesis must be further tested *in vivo* and *in vitro* studies by moderating glycolysis, such as by gene transfer of glycolysis-related genes [33] and/or administration of drugs that activate glycolysis [34].

### Associations among mitochondrial dysfunction, proteostasis, and telomere

Mitochondrial dysfunction due to reactive oxygen species (ROS) has been proposed as one of the determining factors of aging [35]. Inhibition of glucose transporter 1 (Glut1), responsible for a large portion of basal glucose uptake, is known to increase the level of ROS [36]. In contrast, activation of glycolysis induces resistance to ROS [37]. These results may explain the immortality of cancer cells, in spite of aging and cancer share common mitochondrial dysfunction that include an alteration of the mitochondrial genome and activation of mitochondria-to-nucleus signaling pathways [38].

Oxidative stress induces disruption of protein synthesis and degradation, and heat shock proteins (HSP) are known to maintain cellular proteostasis and protect cells from stresses [39]. HSP expression downregulates oxidative phosphorylation, which is the main source of ROS, and upregulates the glycolytic pathway [40]. These findings indicated the significance of glycolysis in the maintenance of cellular proteostasis by HSP. Telomeres are structures at the ends of linear eukaryotic chromosomes and define the proliferation potential of cells. The activation of telomere lengthening mechanisms is coupled with increased proliferation and need for energy resources [41]. During the G1/S cellular phase in which telomerase effects telomere elongation, the glycolytic metabolism program is activated, likely to minimize the risk of oxidative damage to DNA by mitochondria-derived ROS [41]. These findings are consistent with the findings that telomerase is activated in highly glycolytic cells, such as germ, stem, and cancer cells [42].

### Stem cell therapy and glycolysis

This concept introduces a novel therapeutic approach for anti-aging treatments. The mechanism underlying stem cell therapies, including HSC and mesenchymal stem cell transplantation, involves cellular interactions mediated by gap junctions [1, 43]. Gap junctions are specialized intercellular connections composed of connexins that enable the transfer of small, water-soluble molecules, including most metabolites, along concentration gradients [44]. These junctions significantly influence the metabolic status of connected cells [45] and are crucial for development, differentiation, and regeneration [1, 43, 44]. Studies have demonstrated that the intravenous transplantation of HSCs in aged mice enhances glucose transporter transcription, restoring neurological function [46], and activating hippocampal neurogenesis [47]. Moreover, intramuscular HSC injections into ischemic limbs induce significant vascular regeneration in humans [5]. Circulating white blood cells (WBCs) directly interact with endothelium and tissue stem cells through gap junctions [48]. This interaction may explain rejuvenation observed in aged animals by heterochronic parabiosis with young animals [49] and partial bone marrow replacement with young animal bone marrow [50]. These findings suggest multiple pathways to rejuvenate aged humans, indicating that therapeutic strategies can be straightforward [51].

The significant roles of endothelial colony-forming cells (ECFCs), a unique endothelial progenitor subset, are proposed in vascular repair and neurovascular resilience [52]. Aging disrupts ECFCs functionality through oxidative stress, chronic inflammation, and cellular senescence, and priming ECFCs with erythropoietin, that has a potential to activate glycolysis through the activation of Janus kinase 2 (JAK-2) [53], had been shown to improve the function of ECFCs [54]. An emerging non-invasive technique, functional ultrasound (fUS) imaging, provides real-time cerebral blood flow insights in aged brain with notable spatial-temporal resolution [55, 56]. These findings may point to a future direction in treating aging with stem/progenitor cells with objective evaluation methods.

## Conclusion

Our hypothesis that only species with an optimal rate of decline over time survived can explain the four aspects of aging, including most animals do not have an eternal lifespan, lifespans vary considerably among species, why cancer cells can proliferate extensively with abnormal longevity, and why exceptional animals, such as *H. glaber*, do not have an innate lifespan limit. The concept that aging is linked to a decline in glycolytic ATP production, and that species with an optimal rate of decline survive through generation changes, offers new insights into aging science. Although further studies are required to comprehensively understand aging complexities and develop effective treatments, this idea suggests potential strategies for rejuvenating aged individuals by targeting glycolytic ATP production.
